# Influence of Pulsed Electric Field Technology on Functionality and Protein Structure of Evaporated Skim Milk and Nonfat Dry Milk

**DOI:** 10.3390/ijms27083395

**Published:** 2026-04-10

**Authors:** Elizabeth L. Ryan, Owen M. McDougal

**Affiliations:** 1 Biomolecular Sciences PhD Program, College of Arts and Sciences, Boise State University, Boise, ID 83725, USA; elizabethryan611@u.boisestate.edu; 2 Department of Chemistry and Biochemistry, College of Arts and Sciences, Boise State University, Boise, ID 83725, USA

**Keywords:** pulsed electric field, casein micelle, evaporated skim milk, nonfat dry milk, functionality, protein structure

## Abstract

Nonfat dry milk (NFDM) powder was produced by spray drying a pulsed electric field (PEF)-treated solution of 48% (m/m) evaporated skim milk (ESM) that was treated with a field strength of 20 kV/cm and specific energy of 15 kJ/L at 150 L/h. PEF treatment induced reduction to particle size for whey proteins by 8.4% and casein micelles by 11.1% and increased conductivity by 10.6%. The PEF-treated ESM solution was less viscous than the non-PEF control (14.5% lower) and sedimentation was reduced by 40%. Increases to the tapped density (1.9%), solubility (4.7%), and emulsification stability (60%) of the NFDM were observed after PEF treatment. Evaluation of protein structure indicated no modification to the secondary structure, while minor changes to the tertiary structure were observed with increased fluorescence intensity and decreased transition temperatures. The reduction in casein micelle size for the PEF-treated ESM may be associated with the movement of minerals to the aqueous solvent. This study is the first to apply PEF technology to a highly concentrated ESM solution using a continuous flow commercial PEF system. The results of this study suggest that PEF technology may be beneficial to improving the dairy processing efficiency of ESM and product quality for NFDM.

## 1. Introduction

The use of pulsed electric field (PEF) technology to improve the physical and functional properties of dairy solutions has been studied for the past 20 years [1,2,3]. However, the lack of consistency between the studies in PEF systems, parameters, and products gives varying results, whether a positive result or lack thereof, and indications of what benefits may be seen industrially. Furthermore, the use of commercially available systems with industrially achievable application parameters is rare (see Table 1). Most of the research done in this space uses either milk, reconstituted milk, or casein powders. There have been few studies on the effects of PEF treatment on concentrated milk and caseins, through filtration and evaporation, prior to spray drying.

Milk is a complex fluid matrix consisting of macronutrients, such as fats, carbohydrates, and proteins, and micronutrients, including vitamins and minerals, but the vast majority of milk, ~87%, is water [4]. Dairy proteins, which include caseins (80%) and whey (20%), have many positive attributes, including their unique physicochemical properties compared to plant-based protein sources [4,5]. While whey consists of more than 20 water-soluble and globular proteins, caseins exist in micelles, where the casein proteins form a disordered structure with colloidal calcium phosphate [4]. Although fats in milk have many positive effects on human health [6], fats are oftentimes separated from the rest of the milk constituents to create nonfat skim milk. Skim milk can be spray-dried to create easily transportable, shelf-stable milk powders, including skim milk powder (SMP) and nonfat dry milk (NFDM) [4]. SMP and NFDM are similar powders, but SMP is internationally regulated to have a minimum protein level of 34%, whereas NFDM is not regulated but generally has the same protein content. Spray drying is an energy-intensive process that can be improved by introducing concentrated dairy solutions with lower water content, i.e., higher total solids, and/or reduced viscosity, i.e., smaller droplet size providing more surface area and faster powder formation [7].

PEF is a nonthermal food processing technology that alters samples using rapid high-voltage electrical pulses [8]. There are several parameters that can be manipulated to provide optimal treatment to different food materials, including electric field strength, specific energy, pulse width, pulse duration, frequency, and flow rate [8,9]. The companies that make PEF systems include Diversified Technologies, Inc., Pulsemaster B.V., Elea Technology GmbH, Energy Pulse Systems, ScandiNova Systems, and PurePulse Technologies, Inc.; however, research reported in the literature remains primarily dependent on the use of custom-made PEF systems or non-scalable technology [8]. The application of pulsed electric fields can occur in a batch chamber, where a limited volume of sample can be treated, or in a continuous flow treatment cell that permits material to be pumped through the electric field. In the context of PEF system integration into dairy processing, continuous flow systems are required due to the large volume of fluid processed [10]. At present, the potato industry has been the first to implement PEF technology at an industrial scale on the order of 60,000 lbs./h, but other food process industries are keenly interested in the technology to ease cutting of root crops, improve preservation, and extract valuable components, among other advantages [11]. PEF technology could be implemented into dairy processing as liquid systems that can process greater volumes of material are currently being created by several companies. While initial integration may come with high upfront costs, as well as modifications to the processing line, cleaning procedures, and electrical necessities, the systems are relatively small and the benefit has been shown to be economically and environmentally sustainable.

Due to the rising interest in nonthermal technology with the potential for improved processing efficiency and obtaining higher quality products, many industries have experimented to discover the potential benefits of PEF technology, along with ultrasound, irradiation, high pressure, and cold plasma. One such application of PEF is to improve the physical and functional properties of protein-based foods, whether animal- or plant-based, such as dairy, meat, egg, legumes, and cereal proteins [9,12,13,14]. Solubility, emulsification stability, and gelation, along with other functional properties, have been improved through PEF processing. Changes to the inherent physical and functional properties of the proteins have been hypothesized to be due to conformation changes caused by polarization that disrupt inter- and intra-molecular interactions [15].

**Table 1:** Significant changes to physical, functional, and structural properties reported in previous studies using pulsed electric field (PEF) technology with milk or casein proteins. ↑ = increase; ↓ = decrease.

Sample ^a^	PEF System	PEF Parameters ^b^	Results ^c^	Citation
Commercial UHT skim milk	Continuous bench scale PEF System (OSU-3C) at 1 mL/s	35 kV/cm, 64 pulses, 3.0 μs PW, 188 μs TT	No changes to physical properties	[16]
Raw whole milk	PEF system w/a single treatment chamber at 5 L/h	45 kV/cm (500 ns PW) and 55 kV/cm (250 ns PW)	AV ↓ CM PS ↓	[17]
Raw skim milk, reconstituted SMP (10% TS), concentrated skim milk, MPC (18% TS)	OSU-4 lab scale PEF unit with 4 co-field treatment chambers of 0.15 cm diameter at 240 mL/min	45 kV/cm, 2 μs PW, 20 μs TT, 202 kJ/L	AV ↓ (initially)	[18]
Low-heat SMP (5% TS)	TG2 high voltage pulse generator (McGill University) with a treatment chamber with 1.0 cm gap	15–20 kV/cm, 20–60 pulses	FB ↓ AV ↑ CI ↑	[19]
Low-heat SMP (20% m/m)	OSU-4 PEF model with an individual chamber of 1.5 mm diameter at 240 mL/min	42.5–51 kV/cm, 294 J/mL, 19.36 μs TT, 2 μs PW	PS ↑ (high pH)	[20]
Raw whole milk	PEF process design at 4.2 mL/s	20–26 kV/cm, 34 μs TT, 20 μs PW	Protein T_M_ ↓ SH ↑	[21]
MPC 85 (10% TS)	Pilot-scale batch system (Elea) with electrode gap of 10 mm of 86 L/h	4–20 kV/cm, 20 μs PW	II ↓	[22]
Casein micelles	Continuous PEF system HVP 5 (Elea) with 2 colinear treatment chambers at 27 L/h	10–20 kV/cm, 20–100 kJ/kg, 15–25 μs PW	CD & SSA changed SP ↓ β-carotene content ↑	[23]
Casein micelles (1% m/v)	PEF generator (designed in Lithuania) with a 2 mm cuvette	10–30 kV/cm, 10 pulses, 480 ns PD	PS ↓ (higher FS) ZP ↑ FI ↓ (higher FS) β-turn & β-sheet ↑ α-helix & random coil ↓ Disulfide bonds ↑	[24]
MCI (0.25% m/v) in lactose-free simulated milk ultrafiltrate	Colinear treatment chamber with inner diameter of 6 mm at 1 and 5 L/h	16 kV/cm, 2 μs PW, 6 and 31 μs TT (for 1 and 5 L/h), 24–100 kJ/L	FI ↑ CM reorganization	[25]
Commercial MCC (21% TS)	Continuous PEF system (Elea) with electrode with 10 mm gap at 106.7 L/h	4–20 kV/cm, 20 μs PW	AV ↓	[26]

^a^ UHT = ultra-high temperature; SMP = skim milk powder; TS = total solids; MPC = milk protein concentrate; MCI = micellar casein isolate; MCC = micellar casein concentrate; ^b^ PW = pulse width; TT = treatment time; PD = pulse duration; ^c^ PS = particle size; AV = apparent viscosity; CM = casein micelle; II = insolubility index; FS = field strength; ZP = zeta potential; FI = fluorescence intensity; FB = flow behavior; CI = consistency index; CD = charge density; SSA = specific surface area; SP = surface potential; T_M_ = melting temperature; SH = surface hydrophobicity.

Within the dairy industry, PEF has been studied for the inactivation of microorganisms as a potential alternative to traditional pasteurization [10,27,28,29,30,31]. However, with strict compliance guidelines for pasteurization in the dairy industry, this technology is unlikely to gain industrial adoption for that purpose. PEF treatment has also been studied to improve the physical and functional properties of dairy proteins [1,2,3]. Several studies have been conducted with milk and casein solutions, indicating that PEF induces changes to the physical, functional, and structural properties of proteins (see Table 1). In all studies, the only PEF technology manufacturer represented was Elea Technologies. Most of the PEF systems used for scientific research were custom-made and not suitable for application in industrial settings. These studies did report positive effects for PEF-treated milk and casein solutions that included a decreased apparent viscosity, decreased insolubility index, decreased particle size, and increased zeta potential. With regard to protein structure, there were changes induced by PEF to secondary structures, the α-helix, β-sheet, β-turn, and unordered relative content, and the tertiary structure, observed in modified fluorescence intensities, decreased melting temperatures, and casein micelle reorganization.

Upon inspection of the results from prior studies presented in Table 1, there exist numerous discrepancies regarding the impact of PEF treatment on milk and casein-based products. While one study showed no changes to physical properties [16], the rest of the studies showed some changes to the physical, functional, and structural properties. Another discrepancy observed is that certain properties and characteristics, including apparent viscosity, particle size, and fluorescence intensity, were shown to increase through PEF treatment in some studies but decreased in others. There are few studies that investigate the same products or PEF technology. Due to the variability in PEF systems utilized for these studies, the PEF parameters are drastically different, as are the technology capabilities due to pulse generators and treatment chambers. The system differences introduce inconsistencies to results associated with physical and functional property changes for proteins from PEF treatment. Additionally, the previous literature that analyzed structural properties had variable results on micellar casein, namely with the fluorescence and infrared spectroscopy analyses [24,25]; they showed different effects of PEF treatment on fluorescence intensity and infrared spectroscopy analysis yielded statistically different changes in the secondary structure in only one of the studies. While the studies surveyed provide proof of concept using small-scale PEF systems, only one publication references the use of a commercially available, scalable system, with a solution concentrate taken directly from a dairy processing line; the study focused on micellar casein concentrate [26]. The use of both industrially relevant skim milk solutions and commercially available continuous flow PEF systems in the same study is minimal, so it is unknown if positive effects will still be observed with the use of industrially relevant solutions and PEF systems. At present, no studies have been reported for the PEF treatment of evaporated skim milk at 48% (m/m) using a commercially available PEF system at a flow rate of 150 L/h until now.

In the current study, we sought to address the knowledge gap in how using a commercially available, scalable, continuous flow PEF system on concentrated skim milk affects the physical and functional properties of the liquid and powder samples and its effect on the protein structure and casein micelle contents. Evaporated skim milk from a local dairy processor was treated with 20 kV/cm and 15 kJ/L using an Elea PEF Pilot Dual continuous flow system and DN-10 treatment chamber. Following PEF treatment, the evaporated skim milk was spray-dried to create non-PEF-treated (control) and PEF-treated NFDM. The evaporated skim milk was assessed for changes in conductivity, pH, particle size, zeta potential, viscosity, and sedimentation, whereas the powder NFDM was tested for differences in particle size, zeta potential, density, flowability, solubility, foamability, emulsification, and protein molecular weights. Modifications to protein structure were determined through circular dichroism (CD), fluorescence spectroscopy, and differential scanning calorimetry (DSC). Additionally, the mineral and protein content of the casein micelles were measured to determine any changes after PEF treatment. We hypothesize that changes to both the tertiary protein structure and casein micelle contents will decrease the whey protein and casein micelle particle size, which may contribute to positive changes in protein functionality.

## 2. Results and Discussion

### 2.1. Evaporated Skim Milk (Liquid) Physical and Functional Properties

#### 2.1.1. Effect of Pulsed Electric Field on Conductivity and pH

**Figure 1:**
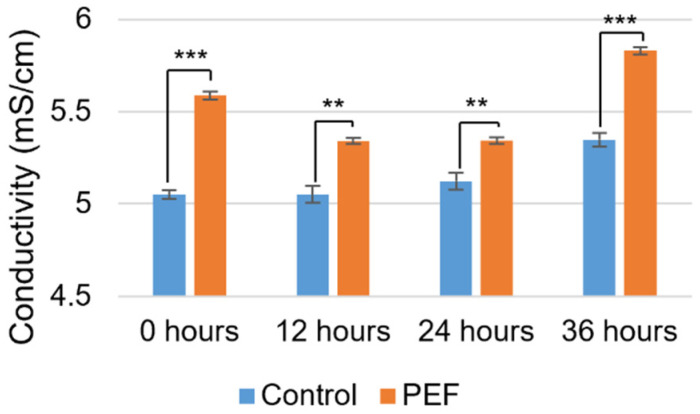
Conductivity (mS/cm) of non-PEF-treated (control) and PEF-treated evaporated skim milk. Measurements represent mean (*n* = 3) and error bars are standard deviation. ** signifies *p* < 0.01 and *** signifies *p* < 0.001 when comparing the PEF-treated measurement to the control.

The conductivity of the evaporated skim milk shows consistently higher levels for PEF-treated samples as compared to the non-PEF control over 36 h (Figure 1). Both the non-PEF-treated control and PEF-treated sample experienced increased conductivity over time, with possible reasons including acidification from lactic acid bacterial activity and casein-bound calcium phosphate release [32]. The increase in conductivity over time for the control sample is only statistically significant (*p* < 0.01) for the 36 h timepoint. The PEF-treated sample significantly (*p* < 0.001) decreases in conductivity from the original test (0 h) to the 12 and 24 h timepoints before significantly (*p* < 0.001) increasing at 36 h. Association of free ions with proteins may occur after a short holding time, but then further dissociation occurs after the longer holding time of 36 h. While the untreated sample conductivity rose from only 5.05 mS/cm to 5.35 mS/cm, the PEF-treated sample had conductivities between 5.35 mS/cm and 5.85 mS/cm. Minerals released into solution may contribute to the increased conductivity after PEF treatment. It may be that the electrical pulses disrupt metal–protein interactions, resulting in metal ions going into solution, and enhanced protein intermolecular interactions with water causing changes to physical and functional properties. The pH was not significantly affected by PEF treatment, maintained between 6.41 and 6.47 throughout the 36 h post-treatment (Figure S1).

#### 2.1.2. Effect of Pulsed Electric Field on Particle Size and Zeta Potential

**Figure 2:**
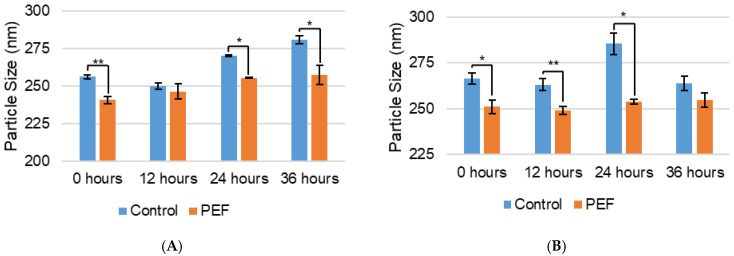
Particle size (nm) of (**A**) whey proteins and (**B**) casein micelles of non-PEF-treated (control) and PEF-treated evaporated skim milk measured by dynamic light scattering (DLS). Measurements represent mean (*n* = 3) and error bars are standard deviation. * signifies *p* < 0.05 and ** signifies *p* < 0.01 when comparing the PEF-treated measurement to the control.

The average particle size of the whey proteins and casein micelles significantly changed due to PEF treatment (Figure 2), as determined by dynamic light scattering (DLS). The particle size distribution can be seen in Figure S2. As shown in Figure 2A, the whey proteins’ size significantly (*p* < 0.01 for control and *p* < 0.05 for PEF) increased after 24 and 48 h in both the control and PEF-treated samples, likely due to agglomeration of the proteins when in solution for a holding time. The average particle size of the whey proteins in the PEF-treated evaporated skim milk was decreased at every time point measured compared to the control, with statistically significant decreases at 0, 24, and 36 h. The relative change in particle size was 6.0, 1.4, 5.4, and 8.4% for 0, 12, 24, and 36 h, respectively. Figure 2B shows that the casein micelle average particle size did not significantly change over time other than the control at 24 h compared to the control initially (*p* < 0.05). The single time point increase may be associated with swelling of the micelle from clustering of the micelles because of the release of calcium phosphate, followed by a decrease to the original size at 36 h from repulsion of the micelles. The average size of the casein micelles was also significantly decreased at 0, 12, and 24 h due to PEF treatment, when compared to the control. The relative changes for 0, 12, 24, and 36 h were 5.8, 5.3, 11.1, and 3.5%, respectively. The results shown here indicate that PEF treatment significantly changes the average particle size of both whey proteins and casein micelles, which may play a role in the effects of PEF on other physical and functional properties. However, the necessary dilutions of the evaporated skim milk before particle size measurement may affect colloidal diffusion or sedimentation and the interactions between individual particles. Evidence of PEF affecting micelle size has been reported in the literature and was attributed to the disruption of bonds that stabilize the casein micelle. PEF treatment of raw skim milk at 45 kV/cm and micellar casein at 10–30 kV/cm showed decreased micelle size [17,24]. Other previous studies with milk concentrates, micellar casein, and commercial UHT skim milk observed no change in particle size from PEF treatment using 45 kV/cm, 16 kV/cm, and 35 kV/cm, respectively [16,18,25]. The contrasting change in particle size between previous studies is likely due to variable PEF treatment and lack of consistency in PEF systems and applied parameters.

The zeta potential was measured for the control and PEF-treated evaporated milk at 0, 12, 24, and 36 h after treatment (Figure S3). The zeta potential was −28 to −32 mV and was not significantly different between the control and PEF-treated evaporated skim milk, indicating that changes in the conductivity of the solution are not due to changes in surface charge measured by zeta potential. While PEF treatment (42.51–51 kV/cm) of reconstituted skim milk also resulted in no change in zeta potential [20], a study on micellar casein demonstrated an increase in the zeta potential after PEF treatment (10–30 kV/cm) [24]. The surface potential absolute value decreased with increasing temperature and PEF treatment of casein micelles, which was attributed to the movement of ions from the micelle surface [23]. Although the zeta potential was not affected in the current study, more intense PEF treatments, higher field strengths and longer pulse widths, may impact protein surface charge.

#### 2.1.3. Effect of Pulsed Electric Field on Viscosity

**Figure 3:**
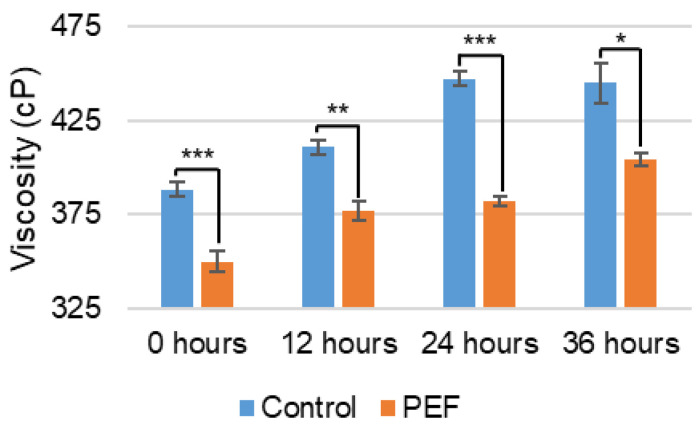
Viscosity (cP) of non-PEF-treated (control) and PEF-treated evaporated skim milk. Measurements represent mean (*n* = 3) and error bars are standard deviation. * signifies *p* < 0.05, ** signifies *p* < 0.01, and *** signifies *p* < 0.001 when comparing the PEF-treated measurement to the control.

Figure 3 shows that the viscosity of the solution naturally increases with time due to age thickening. This increase is statistically significant for both the control (*p* < 0.05) and PEF-treated (*p* < 0.01) samples during the holding period. Apparent viscosity was measured at 0, 12, 24, and 36 h for the untreated control and PEF-treated evaporated skim milk (Figure 3). The PEF-treated evaporated skim milk showed decreased viscosity at every time point compared to the untreated control; as the viscosity of the control sample increased over time, the viscosity of the PEF-treated sample maintained its decreased value compared to the untreated sample, with relative changes in the apparent viscosity after PEF treatment of 9.9, 8.3, 14.5, and 9.2% for 0, 12, 24, and 36 h post-treatment, respectively. The higher decrease in viscosity at 24 h may be associated with the larger decrease observed in casein micelle particle size at the same time. At 12 h, the casein particle size decreases alongside the viscosity but the whey particle size does not, and vice versa at 36 h. Floury et al. (2006) and Raghunath et al. (2024) [17,26] reported that apparent viscosity of raw milk at 45 kV/cm and micellar casein concentrate at 4–20 kV/cm, respectively, could be decreased by application of PEF. Conversely, Xiang, Simpson et al. (2011) [19] found an increase in apparent viscosity of reconstituted skimmed milk and Michalac et al. (2003) [16] found no change in apparent viscosity of raw skim milk when PEF-treated with 15–20 kV/cm and 35 kV/cm, respectively. Although there are some discrepancies across the literature, the consistency with Raghunath et al. (2024) [26] is logical considering similar PEF treatment with the same system and similar field strengths.

#### 2.1.4. Effect of Pulsed Electric Field on Sedimentation

**Figure 4:**
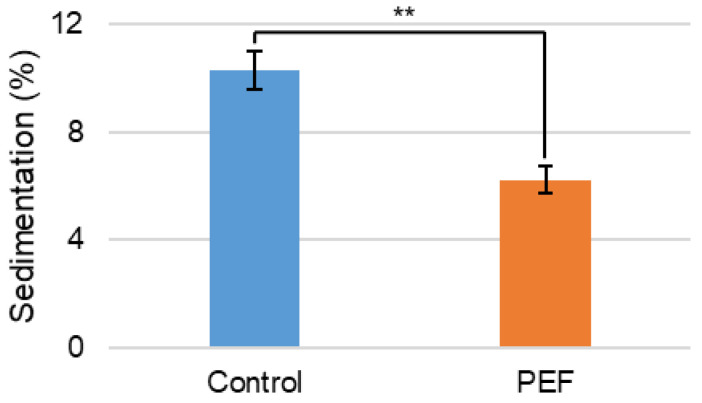
Sedimentation (%) of non-PEF-treated (control) and PEF-treated evaporated skim milk. Measurements represent mean (*n* = 3) and error bars are standard deviation. ** signifies *p* < 0.01 when comparing the PEF-treated measurement to the control.

Sedimentation, or the amount of solid material settling out of the solution, for evaporated skim milk was determined (Figure 4). Sedimentation significantly decreased from about 10% of the initial mass in the non-PEF control, untreated evaporated skim milk, to approximately 6% when PEF-treated. Sedimentation of dairy concentrates prior to spray drying is a significant challenge that appears to be favorably impacted by PEF treatment.

### 2.2. Nonfat Dry Milk (Powder) Functional Properties

#### 2.2.1. Effect of Pulsed Electric Field on Density

The loose density of the non-PEF-treated and PEF-treated NFDM powder were not significantly different; however, the tapped density of the PEF-treated powder was significantly higher than that of the untreated control, amounting to a 1.87% increase (Table 2). The Carr’s index (CI) and Hausner ratio (HR) were also significantly different when compared between the control and PEF-treated. Greater CI and HR values are indicative of a more cohesive powder [33].

#### 2.2.2. Effect of Pulsed Electric Field on Solubility

**Figure 5:**
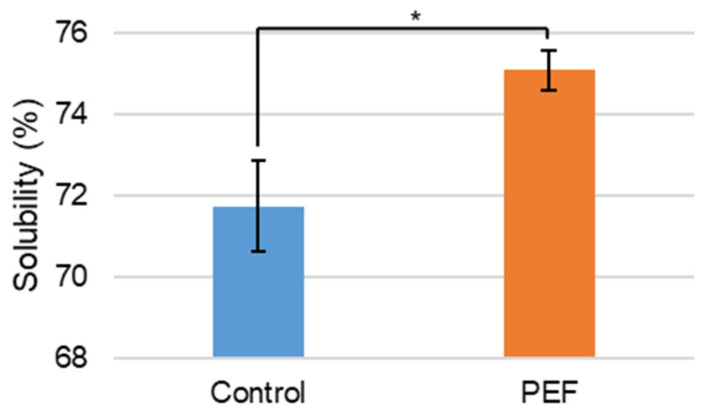
Solubility (%) of non-PEF-treated (control) and PEF-treated NFDM. Measurements represent mean (*n* = 3) and error bars are standard deviation. * signifies *p* < 0.05 when comparing the PEF-treated measurement to the control.

Concentrate solubility was measured for the spray-dried untreated and PEF-treated NFDM. The solubility significantly increased when PEF-treated (Figure 5). The solubility increased to 75.1% when dissolved at 10% (m/m) when PEF-treated compared to the untreated NFDM at 71.7%, accounting for a 4.7% relative change. Increased solubility provides protein products that dissolve into solution more easily. Solubility of protein concentrates may also affect other functional properties, making it a critical property for improvement for subsequent effects, such as the foamability, emulsification, and gelling of protein solutions [9]. Previously, optimized conditions for a batch PEF system to minimize the insolubility index of reconstituted MPC85 were 9.98 kV/cm and 165.43 Hz [26].

#### 2.2.3. Effect of Pulsed Electric Field on Foamability and Emulsification

Foam overrun (FO) portrays a product’s ability to make a foam layer and the foam volume (FV) represents the stability of the foam over time. Figure S4 shows the measured FO and FV for the untreated and PEF-treated reconstituted NFDM, which showed no significant change due to PEF treatment. The FO was determined to be approximately 14 mL/mL, indicating a small foam layer above the liquid (Figure S4A). Approximately 90% of the foam remained after 2 h of sitting, showing great foam stability (Figure S4B).

**Figure 6:**
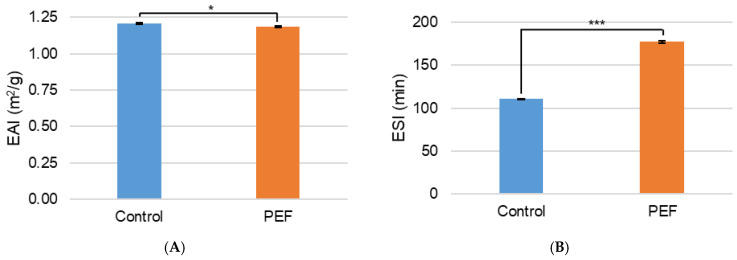
(**A**) Emulsifying activity index (EAI) (m^2^/g) and (**B**) emulsifying stability index (ESI) (min) of non-PEF-treated (control) and PEF-treated NFDM. Measurements represent mean (*n* = 3) and error bars are standard deviation. * signifies *p* < 0.05 and *** signifies *p* < 0.001 when comparing the PEF-treated measurement to the control.

The emulsifying activity index (EAI) tells how well an aqueous solution can form an emulsion with oil and the emulsifying stability index (ESI) explains how long that emulsion is stable. The EAI and ESI of the non-PEF-treated and PEF-treated NFDM are shown in Figure 6. Although the change in EAI was significant between the untreated control and the PEF-treated NFDM, the change was minimal at only a 2% relative change and both samples had an EAI of about 1.2 m^2^/g (Figure 6A). This difference would likely not have any practical significance in product quality and would not be noticeable to consumers. Conversely, the ESI significantly increased due to PEF treatment (Figure 6B). The control NFDM emulsion lasted for 110 min, while the emulsion created by the PEF NFDM stayed for 177 min; this was a relative change in ESI of 60%. Therefore, while PEF minimally affected the EAI of NFDM, it significantly improved the ESI.

#### 2.2.4. Effect of Pulsed Electric Field on Protein Molecular Weights

The molecular weight profile of the untreated control and PEF-treated NFDM are shown in Figure S5. NFDM contains casein and whey proteins, both of which are visible in the SDS-PAGE gel. The two main caseins, α-casein and β-casein, are present with molecular weights of approximately 24 and 25 kDa, respectively. Additionally, the two most abundant whey proteins, α-lactalbumin and β-lactoglobulin, can be seen with molecular weights of approximately 14 and 18 kDa, respectively. The results indicate that PEF treatment does not impact protein molecular weights for the most abundant dairy proteins in NFDM. Liu et al. (2015) [20] used PEF with reconstituted skim milk at higher field strengths of 42.5 to 51 kV/cm and saw no change in individual milk proteins, consistent with the findings of the current study and much of the literature precedence regarding PEF-treated proteins [12].

### 2.3. Structural Properties

#### 2.3.1. Effect of Pulsed Electric Field on Secondary Structure

Protein secondary structure elements can be determined using circular dichroism (CD) [34]. The CD spectra of the untreated and PEF-treated NFDM are very similar, with only the PEF-treated spectrum having a larger negative dip at approximately 202 nm (Figure S6). These spectra are indicative of a protein that has a large amount of random coil, or unordered regions [34], which is likely because of the unordered nature of casein proteins [35]. Using DichroWeb for deconvolution [36,37], the percentage of each secondary structure element was estimated (Table 3). The estimations support that the most abundant secondary structure element is the unordered regions. The deconvolution also provided information on the difference in secondary structures between the control and PEF-treated NFDM, finding that there was no significant difference. Therefore, under the conditions of the present study, PEF treatment did not disrupt the protein secondary structure. Similarly, Morais et al. (2023) [25] observed no change in secondary structure elements when using PEF treatment of 16 kV/cm on micellar casein isolate. Conversely, Taha et al. (2023) [24] observed the unfolding of casein micelles and significant changes in secondary structure elements, attributed to decreases in the α-helix and random coil, and increases to the β-turn and β-sheet, when PEF-treating micellar casein at 10 to 30 kV/cm.

#### 2.3.2. Effect of Pulsed Electric Field on Tertiary Structure

**Figure 7:**
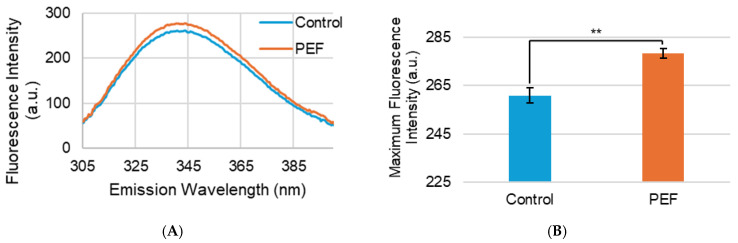
(**A**) Fluorescence spectra and (**B**) maximum fluorescence intensity of non-PEF-treated (control) and PEF-treated NFDM. Measurements represent mean (*n* = 3) and error bars are standard deviation. ** signifies *p* < 0.01 when comparing the PEF-treated measurement to the control.

Modifications to protein tertiary structure can be assessed with the use of intrinsic fluorescence spectroscopy, which measures natural fluorescence emitted from aromatic, hydrophobic amino acids, such as tryptophan [38,39]. The fluorescence spectra of the untreated control and PEF-treated NFDM are shown in Figure 7A. The emission spectra look similar to each other, with the exception of the PEF curve having higher intensity than that of the control. This is also apparent in Figure 7B, which shows the maximum fluorescence intensities of the control and PEF-treated NFDM. The PEF-treated sample has a statistically significant increase in fluorescence intensity that indicates that PEF treatment causes the milk proteins to disassociate slightly to allow hydrophobic residues, especially tryptophan with its high extinction coefficient, to become exposed. Similarly, Morais et al. (2023) [25] observed an increase in fluorescence intensity from tryptophan and tyrosine due to PEF treatment of micellar casein isolate at 16 kV/cm and 4 °C. Another study that used PEF to treat micellar casein resulted in an increase in fluorescence intensity at lower field strengths of 10 kV/cm that decreased when the field strength was raised above 10 kV/cm [24], indicating that PEF may cause conformation alterations to milk proteins dependent on treatment levels.

#### 2.3.3. Effect of Pulsed Electric Field on Thermal Properties

Differential scanning calorimetry (DSC) can be utilized to determine protein thermal properties [40]. Figure S7 shows the thermograms of the untreated control and PEF-treated NFDM powders, where there is an obvious difference between the peaks, indicated by arrows, in the two thermograms. The transition temperatures, determined by the peaks, can be found in Table 4. Each of the transition temperatures for the PEF-treated samples is lower than the untreated control samples. The first two transition points are statistically significant between the control and PEF samples, showing that PEF significantly influences the thermal properties of milk proteins. Likewise, with increased intensity of PEF treatment when using field strengths of 20 to 26 kV/cm, Sharma et al. 2016 [21] observed denaturation of dairy proteins, both whey and casein.

### 2.4. Casein Micelles

#### 2.4.1. Effect of Pulsed Electric Field on Mineral Content

**Figure 8:**
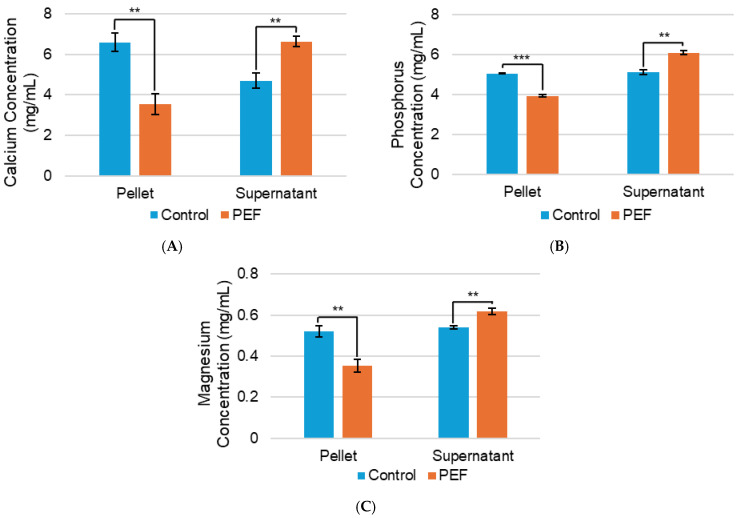
Concentrations of (**A**) calcium, (**B**) phosphorus, and (**C**) magnesium of the pellet (casein micelle) and supernatant (surrounding aqueous solution) of non-PEF-treated (control) and PEF-treated evaporated skim milk. Measurements represent mean (*n* = 3) and error bars are standard deviation. ** signifies *p* < 0.01 and *** signifies *p* < 0.001 when comparing the PEF-treated measurement to the control.

Ion concentration is significant to the conductivity of a solution, so inductively coupled plasma-mass spectrometry (ICP-MS) was used to measure the mineral content of casein micelles and surrounding aqueous solution (Figure 8). Calcium and phosphorus were analyzed because of their stabilizing role in casein micelles as calcium phosphate [35] and magnesium was chosen due to its connection to protein stabilization [41]. Figure 8A shows that the calcium concentration was lower in the casein micelle for the PEF-treated evaporated skim milk than the untreated control, and the concentration of calcium in the surrounding aqueous solution increased with PEF treatment, compared to the control. The same effect was observed for phosphorus and magnesium in Figure 8B and Figure 8C, respectively. The measured values are listed in Table S1. These results imply that PEF treatment may cause minerals to move from the casein micelle to the surrounding solution. The higher calcium, phosphorus, and magnesium presence in the solution may explain the increased solution conductivity and decreased casein micelle particle size after PEF treatment.

#### 2.4.2. Effect of Pulsed Electric Field on Protein Content

**Figure 9:**
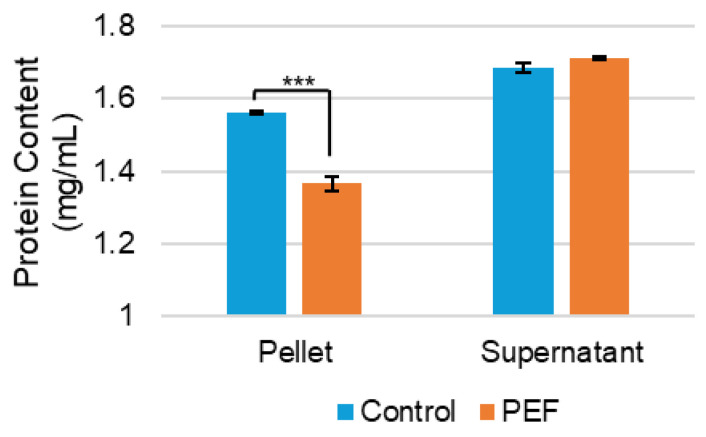
Protein content (mg/mL) of the pellet (casein micelle) and supernatant (surrounding water) of non-PEF-treated (control) and PEF-treated evaporated skim milk. Measurements represent mean (*n* = 3) and error bars are standard deviation. *** signifies *p* < 0.001 when comparing the PEF-treated measurement to the control.

Casein proteins are the main component of casein micelles [35]. The protein content of the micelle and surrounding water was measured by Bradford assay, which showed that the protein concentration in the casein micelles decreased following PEF treatment (Figure 9). There was a slight, statistically insignificant increase in the aqueous phase’s protein content of the PEF-treated evaporated milk after PEF treatment. Therefore, it cannot be definitively determined that protein moves from the casein micelle to the surrounding liquid. Additionally, gel electrophoresis was employed to observe the specific casein proteins in the micelle and surrounding solution (Figure S8). There was a slight increase in casein concentration in the solution after PEF treatment (lane 4 versus lane 5). The Bradford assay and gel electrophoresis results do not provide evidence that the protein content in the casein micelles changes due to PEF treatment.

## 3. Materials and Methods

### 3.1. Sample Collection and PEF Treatment

#### 3.1.1. Sample Collection

Low-heat evaporated skim milk (10 gal) was collected from a local dairy facility directly from the processing line prior to the spray dryer. The facility determined the solids content to be 47.83% with a solid analyzer (SMART6, CEM Corporation, Matthews, NC, USA). The sample’s initial conductivity and pH were 5.05 mS/cm and 6.42, respectively. The sample was transported on ice and PEF-treated within 5 h, not surpassing room temperature (23 °C).

#### 3.1.2. Pulsed Electric Field Treatment

PEF treatment was conducted using a PEF Pilot Dual system (Elea Technologies GmbH, Quakenbrück, Germany) equipped with a DN-10 flow cell (10 mm internal diameter electrode and chamber volume of 1.57 cm^3^). The temperature of the evaporated skim milk was maintained at or below room temperature (23 °C) during the whole process and pumped (progressive cavity pump, INOXPA Kiber KSFT, Plano, TX, USA) through the PEF system at a flow rate of 150 L/h. A non-PEF control sample was acquired by running the evaporated skim milk through the PEF system with the generator off. PEF treatment consisted of monopolar square pulses with the following parameters: field strength of 20 kV/cm, specific energy of 15 kJ/L, pulse frequency of 39 Hz, pulse width of 6 μs, pulse current of 130 A, residence time of 0.038 s, and treatment time of 8.82 µs. The PEF parameters for the current study were derived from a survey of the literature-reported PEF conditions. We conducted 24 experiments with unique PEF parameter combinations (Table S2), using 10% m/m reconstituted SMP, to broaden the literature-stated treatment parameters that included field strengths over the range of 10–21 kV/cm and specific energies of 8–30 kJ/L. The study focused on identifying the field strength and specific energy that provided optimal results associated with sedimentation, foamability, and emulsification stability for the PEF-treated solution immediately following treatment and after 24 h. The result was a field strength of 20 kV/cm and a specific energy of 15 kJ/L. Certain liquid analyses were measured at time points of 0, 12, 24, and 36 h to represent the evaporated skim milk being held in a storage silo before spray drying. During this holding period, the sample was stored in a refrigerator at 4 °C to prevent microbial growth and spoilage.

The evaporated skim milk was spray-dried using a mini spray dryer (USA Lab, Livonia, MI, USA) with the inlet temperature set to 180 °C, and a flow rate that was adjusted to maintain an outlet temperature between 80 °C and 90 °C. A moisture analyzer (Model: MB120, OHaus Corporation, Parsippany, NJ, USA) set to a fast method and final temperature of 80 °C determined the NFDM moisture content to be less than the 4.0%, which is within United States regulatory limits [42].

### 3.2. Physical and Functional Properties (Liquid)

#### 3.2.1. Conductivity and pH

Conductivity and pH were measured immediately after PEF treatment (0 h) and 12, 24, and 36 h post-treatment. Conductivity was measured using a Fisherbrand™ Traceable™ conductivity meter pen (Thermo Fisher Scientific Inc., Waltham, MA, USA). The solution pH was measured with a benchtop pH meter (Fisherbrand™ accumet™ AE150, Thermo Fisher Scientific, Inc., Waltham, MA, USA). Measurements were taken in triplicate.

#### 3.2.2. Particle Size and Zeta Potential

The particle size of whey protein and casein micelles in evaporated skim milk were measured by dynamic light scattering (DLS) with a ZetaSizer Pro (Malvern Panalytical Ltd., Malvern, UK) immediately after PEF treatment (0 h) and 12, 24, and 36 h post-treatment. The liquid was diluted 1:500 before 1.0 mL was pipetted into a polystyrene cuvette (Thermo Fisher Scientific Inc., Waltham, MA, USA) with a pathlength of 10 mm. The instrument has a detection range of 0.6 to 10,000 nm. The instrument was equilibrated to 25 °C for 30 s prior to measurement after the cuvette was loaded. The refractive indices of 1.46 and 1.58 were used to measure whey proteins and casein micelles, respectively [43]. The ZS XPLORER software (Version: 4.0.0.683) was used to determine mean particle size (z-average) and the polydispersity index (PDI) with a general-purpose analysis model. Measurements were performed in triplicate for both whey protein and casein micelle size.

The zeta potential was determined using the same diluted sample in folded capillary zeta cells (Malvern Panalytical Ltd., Malvern, UK) at the same time points as particle size. The instrument was calibrated to 25 °C for 120 s after the cuvette was loaded. Between 10 and 50 scans were recorded per sample with at least 60 s between each acquisition. The monomodal analysis model determined zeta potential and measurements were taken in triplicate.

#### 3.2.3. Viscosity

Apparent viscosity was determined using a cone/plate rheometer (Model: LV-DVNext, AMETEK Brookfield Engineering Inc., Middleboro, MA, USA) immediately after PEF treatment (0 h) and 12, 24, and 36 h post-treatment. The evaporated skim milk was kept in an ice bath at 0 °C to ensure consistent temperature throughout the experiment and holding conditions (4 °C), in line with some processing facilities. The sample (0.5 mL) was pipetted into the cone and the system was run at 0.25 rpm for 15 s. The endpoint viscosity (cP) was recorded, along with the torque (%), in triplicate.

#### 3.2.4. Sedimentation

Evaporated skim milk (100 mL) was stirred on a stir plate at 350 rpm to ensure homogenization before three centrifuge tubes were filled with 20 g of sample each for triplicate measurements, with the mass being recorded. The sample was centrifuged (Centrifuge 5920R, Rotor FA-6x250, Eppendorf GmbH, Wesseling-Berzdorf, Germany) at 15,000 rcf for 20 min. The supernatant was discarded and the pellet was dried (FreeZone 2.5 Plus, Labconco Corp., Kansas City, MO, USA) and massed. Sedimentation was calculated according to Equation (1) [44], where *SP* is the sedimented pellet mass and *I* is the initial mass.

(1) $$Sedimentation\;\left(\%\right)=\frac{SP}{I}\times 100\%,$$

### 3.3. Physical and Functional Properties (Powder)

#### 3.3.1. Density

The density was analyzed using the method described in Pugliese et al. (2017) [45]. Loose density was determined by loosely filling a 10 mL glass graduated cylinder to 10 mL and determining the mass of the powder according to Equation (2). The tapped density was determined by lightly tapping the graduated cylinder 3 times per second from a height of 1 cm on the countertop for 10 min. The new “tap” volume was recorded and the tapped density determined according to Equation (3). The powder flowability with Carr’s index (CI) and cohesiveness with the Hausner ratio (HR) were calculated using Equations (4) and (5), respectively. Measurements were taken in triplicate.

(2) $$Loose\;density=\frac{powder\;mass\;\left(g\right)}{powder\;volume\;\left(mL\right)},$$

(3) $$Tapped\;density=\frac{powder\;mass\;\left(g\right)}{tapped\;powder\;volume\;\left(mL\right)},$$

(4) $$CI=\frac{tapped\;density-loose\;density}{tapped\;density}\times 100,$$

(5) $$HR=\frac{tapped\;density}{loose\;density},$$

#### 3.3.2. Solubility

Solubility was determined using a protocol adapted from Melchior et al. (2020) [44]. The NFDM was massed and reconstituted to 10% (m/m) in nanopure water and shaken (Thermo Scientific Solaris Open Air Orbital Shaker, Thermo Fisher Scientific Inc., Waltham, MA, USA) for 1 h at 350 rpm. The solution was then centrifuged (Centrifuge 5920R, Rotor FA-6x250, Eppendorf GmbH, Wesseling-Berzdorf, Germany) for 20 min at 15,000-rcf. The supernatant was discarded and the insoluble fraction was freeze-dried (FreeZone 2.5 Plus, Labconco Corp., Kansas City, MO, USA) overnight. The dry pellet was massed and the solubility was calculated with Equation (6), where *S* represents the initial sample mass and *DIF* represents the dried insoluble fraction mass. The solubility was determined in triplicate.

(6) $$Solubility\;\left(\%\right)=\frac{S-DIF}{S}\times 100\%,$$

#### 3.3.3. Foamability and Emulsification

As previously described in Schmidt et al. (2018) [46] and Hammershøj et al. (2004) [47], a 1 g/L NFDM solution was hand-shaken for 45 s at 4 Hz in a centrifuge tube. Visual measurements of liquid and foam volumes immediately after the foam preparation were used to determine foam overrun using Equation (7). The foam was left undisturbed for 2 h and the volume of liquid and foam was recorded at 0, 5, 15, 30, 60, 90, and 120 min. The foam volume (FV) was calculated with Equation (8). The foam properties were measured in triplicate.

(7) $$FO\;\left(\frac{mL}{mL}\right)=\frac{V_{foam}}{V_{liquid}},$$

(8) $$FV\;\left(\%\right)=\frac{V_{foam\;\left(t=120\;min\right)}}{V_{foam\;\left(t=0\;min\right)}}\times 100\%,$$

Using a method adjusted from Khalesi & FitzGerald (2021) [48], the emulsifying properties of NFDM samples reconstituted to 0.05 g/mL at pH 7 were determined. The samples were solubilized in a water bath (Precision SWB 15, Thermo Fisher Scientific Inc., Waltham, MA, USA) for 30 min at 50 °C while shaking at 30 rpm. Commercial sunflower oil was used as the oil phase in a 1:3 ratio with protein suspension [46]. The emulsion was created using a Fisherbrand 850 Homogenizer (Thermo Fisher Scientific Inc., Waltham, MA, USA) for 1 min at 16,000 rpm. An aliquot was obtained from the center of the emulsion layer and stabilized with 0.1% sodium dodecyl sulfate (SDS) in a 1:40 ratio before being vortexed for 30 s. The turbidity was determined by measuring the absorbance at 500 nm in a quartz cuvette (Type 9 Semi-Micro, FireflySci, Brooklyn, NY, USA) on a BioTek Epoch 2 Microplate Spectropolarimeter (Agilent Technologies Inc., Santa Clara, CA, USA) [46,49,50,51,52]. The emulsifying activity index (EAI) and emulsifying stability index (ESI) were calculated using Equations (9) and (10) [46], respectively, where *A_0_* represents initial absorbance, *N* represents the dilution factor with SDS (40), *c* represents the concentration in g/mL (0.05), *φ* represents the oil volume fraction (0.25), *A*_30_ represents the absorbance at 30 min, and *t* represents time (30).

(9) $$EAI\;\left(\frac{m^{2}}{g}\right)=\frac{2\times 2.303\times A_{0}\times N}{c\times \varphi \times 10,000},$$

(10) $$ESI\;\left(min\right)=\frac{A_{0}}{A_{0}-A_{30}}\times t,$$

#### 3.3.4. Gel Electrophoresis

Molecular weights of the proteins present in reconstituted NFDM at 1% and 0.5% (m/m) were qualitatively observed with sodium dodecyl sulfate polyacrylamide gel electrophoresis (SDS-PAGE). A 2× Laemmli sample buffer (5% β-mercaptoethanol) was added to each sample in a 2:1 ratio and allowed to sit at room temperature (~20 °C) for 1 h before heating at 95 °C for 5 min. The pre-cast gel (4–20% Mini-PROTEAN^®^ TGX™ Precast Protein Gel, Bio-Rad Laboratories Inc., Hercules, CA, USA) was loaded with 10 μL of sample and protein ladder (Precision Plus Protein All Blue Standards, Bio-Rad Laboratories Inc., Hercules, CA, USA) into their own lanes. The gel was run on a Mini-PROTEAN^®^ Tetra Vertical Electrophoresis Cell (Bio-Rad Laboratories Inc., Hercules, CA, USA) for 1 h at 150 V, or until the dye front reached the bottom of the gel. Gels were stained with Coomassie Brilliant Blue R-250 Staining Solution (Bio-Rad Laboratories Inc., Hercules, CA, USA) while shaking at 30 rpm, destained (10% acetic acid, 40% methanol, 50% water) overnight while shaking at 30 rpm, and imaged the next morning on a Protein Simple FluorChem E system (BioTechne Corp., Minneapolis, MN, USA).

### 3.4. Structural Properties

#### 3.4.1. Circular Dichroism

A J-1500 circular dichroism (CD) spectrometer (Jasco Corp., Tokyo, Japan) was used to collect the CD spectra of NFDM reconstituted to 0.5 mg/mL. Spectra were collected over a wavelength range of 180–270 nm with a bandwidth of 0.1 nm in a quartz cuvette with a pathlength of 0.1 cm. The spectra were collected in triplicate. The secondary structure content, α-helix, β-sheet, β-turn, and unordered, were determined by deconvolution with the DichroWeb website (http://dichroweb.cryst.bbk.ac.uk/html/home.shtml, accessed on 15 December 2025) [53] using the algorithm CDSSTR [54] with Reference Set 6 [55].

#### 3.4.2. Fluorescence Spectroscopy

Intrinsic fluorescence was determined with a Cary Eclipse spectrofluorometer (Agilent Technologies Inc., Santa Clara, CA, USA). NFDM was reconstituted to 0.5 mg/mL and pipetted into a microcell triangular quartz cuvette consisting of a square base and 10 mm pathlength to accommodate front-face fluorescence. Triplicate spectra were collected over an emission wavelength range of 305–400 nm with an excitation wavelength of 290 nm [56,57,58]. Other parameters included excitation and emission slit widths of 5 nm, a scan rate of 60 nm/min, a data interval of 0.5 nm and an averaging time of 0.5 s.

#### 3.4.3. Differential Scanning Calorimetry

Melting temperatures were determined with a STARe Differential Scanning Calorimetry (DSC) 3 system (Mettler-Toledo International Inc., Columbus, OH, USA) with the STARe software (V18.00a). An indium standard with a melting point of 156.6 °C was used to calibrate the instrument [59]. About 10 mg of NFDM powder were put in a pan, sealed, and placed inside the instrument with an empty reference pan. Samples were heated from 30 to 140 °C. Thermograms were collected in triplicate.

### 3.5. Casein Micelles

#### 3.5.1. Separation of Casein Micelles

Evaporated skim milk (48% m/m) was separated using centrifugation (adapted from Pranata et al., 2024) [60], by equilibrating the sample and centrifuge rotor to 4 °C, putting approximately 20 g of sample into a centrifuge tube, and centrifuging (Centrifuge 5920R, Rotor FA-6x250, Eppendorf GmbH, Wesseling-Berzdorf, Germany) at 20,000 rcf for 2 h at 4 °C. About 5 g of supernatant was collected from the top and placed in a separate tube. The sample underwent two more rounds of centrifugation with collection of the supernatant after each to yield two samples—pellet (casein micelles) and supernatant (aqueous solution).

#### 3.5.2. Mineral Analysis

The pellet and supernatant for the non-PEF control and PEF-treated evaporated skim milk were acid-digested using a method adapted from “EPA Method 3050B (SW-846): Acid Digestion of Sediments, Sludges, and Soils” to isolate inorganic matter prior to inductively coupled plasma mass spectrometry (ICP-MS). Digested samples were further diluted with 5% nitric acid at 1:100, respectively. Minerals were quantified using a 7850 ICP-MS (Agilent Technologies Inc., Santa Clara, CA, USA). Mineral content was determined in triplicate.

#### 3.5.3. Protein Content

A Bradford assay was performed on untreated and PEF-treated pellets and supernatants to quantify protein content. A seven-point calibration curve was created with serial dilutions of bovine serum albumin (BSA). The sample (30 μL) was mixed with 1.5 mL of Coomassie Brilliant Blue G-250 (Thermo Fisher Scientific, Waltham, MA, USA) in a plastic cuvette (MilliporeSigma, Burlington, MA, USA) and incubated at room temperature for 10 min. The absorbance was determined at 595 nm and protein content was quantified from the calibration curve. Measurements were taken in triplicate.

Qualitative analysis of the proteins in the pellets and supernatants was performed by tris-tricine gel electrophoresis. Samples (3% m/m) were mixed with tricine sample buffer (5% β-mercaptoethanol) in a 1:1 ratio and left at room temperature (~20 °C) for 1 h before heating at 70 °C for 10 min. The gel (16.5% Mini-PROTEAN^®^ Tris-Tricine pre-cast gels, Bio-Rad Laboratories Inc., Hercules, CA, USA) was loaded with 10 μL of each sample and a protein standard ladder (Precision Plus dual Xtra Protein Standards, Bio-Rad Laboratories Inc., Hercules, CA, USA). The gels were run in a Mini-PROTEAN^®^ Tetra Vertical Electrophoresis Cell (Bio-Rad Laboratories Inc., Hercules, CA, USA) at 100 V for 2 h, or until the dye front reached the bottom of the gel. The gels were fixed (10% acetic acid, 40% methanol, 50% water) for 30 min, stained with Coomassie Brilliant Blue R-250 Staining Solution (Bio-Rad Laboratories Inc., Hercules, CA, USA) for 30 min, and destained (10% acetic acid, 40% methanol, 50% water) overnight, all while being shaken at 30 rpm. The gel was imaged on a Protein Simple FluorChem E system (BioTechne Corp., Minneapolis, MN, USA).

### 3.6. Statistical Analysis

All experiments were performed in triplicate using three distinct samples and results represent the mean ± standard deviation. Error bars express the standard deviation for triplicate measurement in each graph. Student’s *t*-test was used to determine statistical significance with a 95% confidence interval, so *p* < 0.05 of a PEF-treated sample compared to the control was significant. Relative change was determined using Equation (11).

(11) $$Relative\;change\;\left(\%\right)=\frac{PEF-Control}{Control}\times 100\%,$$

## 4. Conclusions

This study is the first to apply PEF technology to a highly concentrated, evaporated skim milk (ESM) solution using a continuous flow commercial PEF system. The results show that PEF can affect some physical and functional properties of ESM and NFDM, such as increased conductivity, tapped density, solubility, and emulsification stability and reduced casein micelle and whey protein particle size, apparent viscosity, and sedimentation. The work reveals slight changes in tertiary structure and movement of minerals out of the casein micelle, which may be associated with improved physical and functional properties. Although this study does not directly show improvement to dairy protein powder production efficiency, decreased apparent viscosity and sedimentation may improve the solution’s properties during steps in the processing line, while the increased tapped density and solubility would improve some aspects of the final powder product for consumers. This study was limited by diluted samples for certain analyses that are necessary for instrumentation but then fail to show the interactions of individual particles and a single PEF condition for this specific evaporated skim milk product. This evaporated skim milk from the local dairy processor may not be processed the same as other processors, leading to differences in the overall product. Future research should explore the scalability of this process, shelf-life stability, direct processing efficiency, and sensory evaluation of the ESM and NFDM. Overall, this study shows the positive effect on some physical and functional properties of a concentrated ESM product with a commercial, continuous flow PEF system.

## 5. Patents

A patent has been filed on the continuous flow process of PEF applied to dairy solutions on 16 September 2025 and assigned Provisional Patent Application no: 63/882,555, titled “Continuous Flow Pulsed Electric Field (PEF) Treatment of Dairy Solutions.”

## Figures and Tables

**Table 2:** Loose density, tapped density, Carr’s index (CI), and Hausner ratio (HR) of powder non-PEF-treated (control) and PEF-treated NFDM. Measurements represent mean ± standard deviation (*n* = 3).

Treatment	Loose Density (g/mL)	Tapped Density (g/mL)	Carr’s Index (CI) (%)	Hausner Ratio (HR)
Control	0.586 ± 0.003	0.697 ± 0.002	15.8 ± 0.6	1.19 ± 0.01
PEF	0.581 ± 0.004	0.712 ± 0.004 *	18.2 ± 0.2 *	1.22 ± 0.01 *

* signifies *p* < 0.05 when comparing the PEF-treated measurement to the control.

**Table 3:** Secondary structure content of non-PEF-treated (control) and PEF-treated NFDM. Measurements represent mean ± standard deviation (*n* = 3).

Treatment	Secondary Structure Content (%)			
	Helix	Strand	Turn	Unordered
Control	3.0 ± 0.0	28.0 ± 1.6	19.7 ± 0.9	47.3 ± 2.6
PEF	3.7 ± 0.5	29.3 ± 0.5	17.3 ± 0.9	48.3 ± 0.5

**Table 4:** Transition temperatures (°C) of non-PEF-treated (control) and PEF-treated NFDM. Measurements represent mean ± standard deviation (*n* = 3).

Treatment	Transition Temperature (°C)		
	Peak 1	Peak 2	Peak 3
Control	52.2 ± 0.1	62.1 ± 0.2	121.2 ± 1.1
PEF	41.9 ± 0.7 **	53.8 ± 0.2 ***	115.6 ± 2.5

** signifies *p* < 0.01 and *** signifies *p* < 0.001 when comparing the PEF-treated measurement to the control.

## Data Availability

The original contributions presented in this study are included in the article/Supplementary Material. Further inquiries can be directed to the corresponding author.

## Supplementary Materials

The following supporting information can be downloaded at: https://www.mdpi.com/article/10.3390/ijms27083395/s1.

## Abbreviations

The following abbreviations are used in this manuscript:

NFDM	Nonfat dry milk
PEF	Pulsed electric field
ESM	Evaporated skim milk
kV/cm	Kilovolt per centimeter
kJ/L	Kilojoule per liter
SMP	Skim milk powder
UHT	Ultra-high temperature
PW	Pulse width
TT	Treatment time
TS	Total solids
MPC	Milk protein concentrate
PD	Pulse duration
MCI	Micellar casein isolate
MCC	Micellar casein concentrate
CD	Circular dichroism
DSC	Differential scanning calorimetry
CI	Carr’s index
HR	Hausner ratio
FO	Foam overrun
FV	Foam volume
EAI	Emulsifying activity index
ESI	Emulsifying stability index
SDS-PAGE	Sodium dodecyl sulfate-polyacrylamide gel electrophoresis
ICP-MS	Inductively coupled plasma-mass spectrometry
DLS	Dynamic light scattering
PDI	Polydispersity index
BSA	Bovine serum albumin
