# Supplementary material for: Influence of Pulsed Electric Field Technology on Functionality and Protein Structure of Evaporated Skim Milk and Nonfat Dry Milk
Source: Int J Mol Sci. 2026 Apr 10;27(8):3395. doi: 10.3390/ijms27083395 (PMC13116837; doi:10.3390/ijms27083395)
Supplement: Supplementary file 1 [file ijms-27-03395-s001.zip › ijms-4226379-supplementary.pdf]

International Journal of
Molecular Sciences

MDPI

Article

Influence of Pulsed Electric Field Technology on Functionality and Protein Structure of Evaporated Skim Milk and Nonfat Dry Milk

Elizabeth L. Ryan [1] and Owen M. McDougal [2,*]

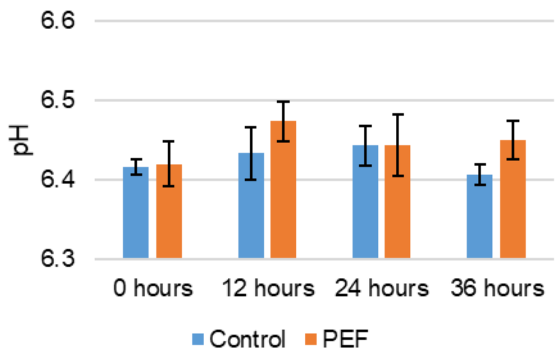

Figure S1. pH of non-PEF-treated (control) and PEF-treated evaporated skim milk. Measurements represent mean (*n*=3) and error bars are standard deviation.

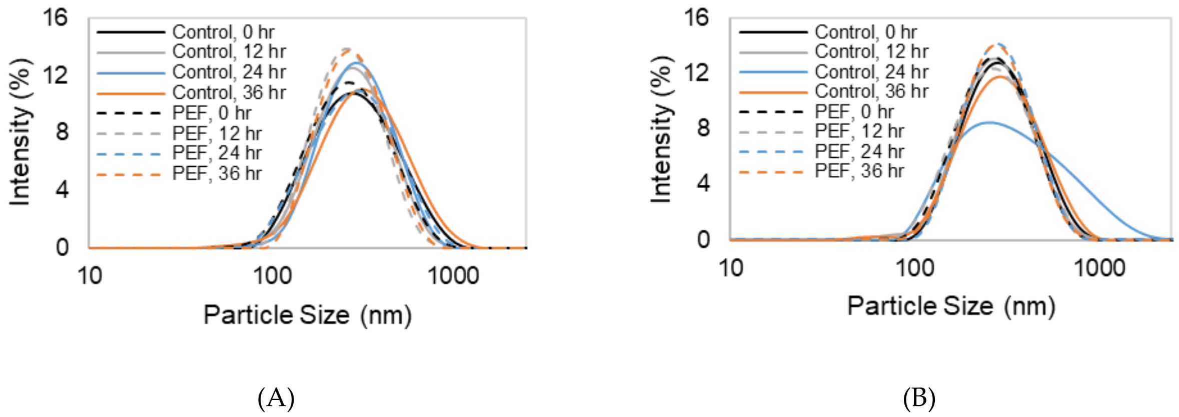

Figure S2. Intensity of particle size distribution of (A) whey proteins and (B) casein micelles of non-PEF-treated (control) and PEF-treated evaporated skim milk. Measurements represent mean (*n*=3).

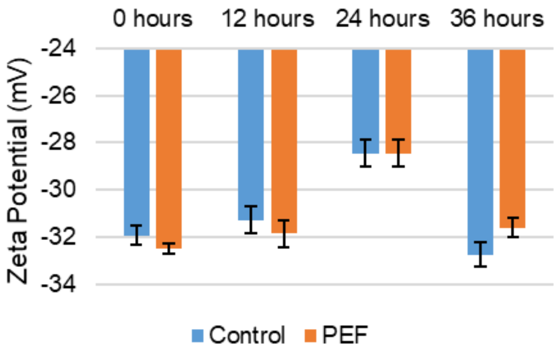

Figure S3. Zeta potential (mV) of non-PEF-treated (control) and PEF-treated evaporated skim milk. Measurements represent mean (n=3) and error bars are standard deviation.

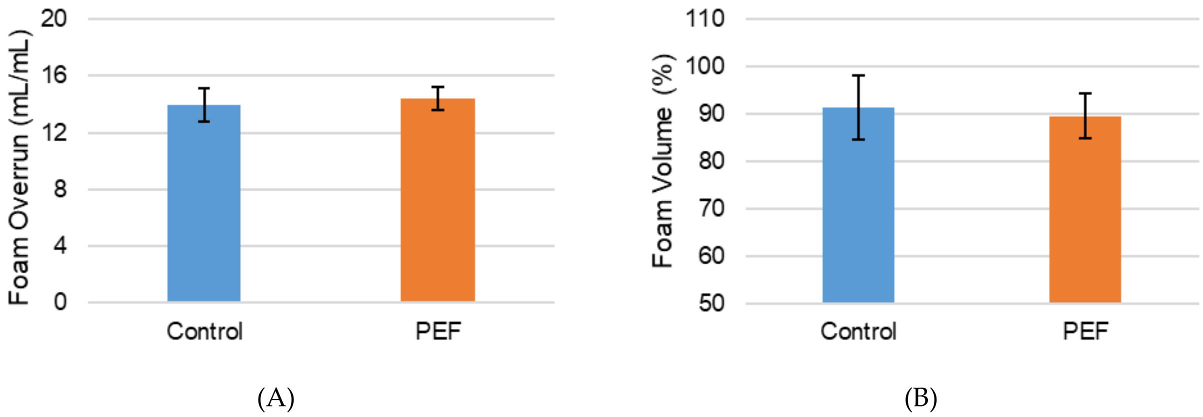

Figure S4. (A) Foam overrun (mL/mL) and (B) foam volume (%) of non-PEF-treated (control) and PEF-treated NFDM. Measurements represent mean (n=3) and error bars are standard deviation.

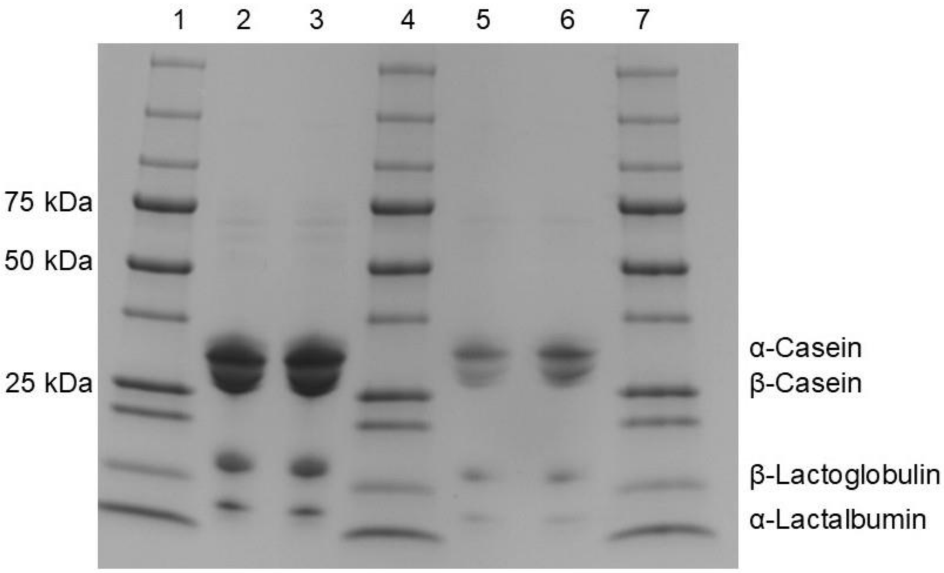

Figure S5. Molecular weight profile of non-PEF-treated (control) and PEF-treated NFDM. Lanes: 1 – protein standard ladder; 2 – control (1%); 3 – PEF (1%); 4 – protein standard ladder; 5 – control (0.5%); 6 – PEF (0.5%); 7 – protein standard ladder.

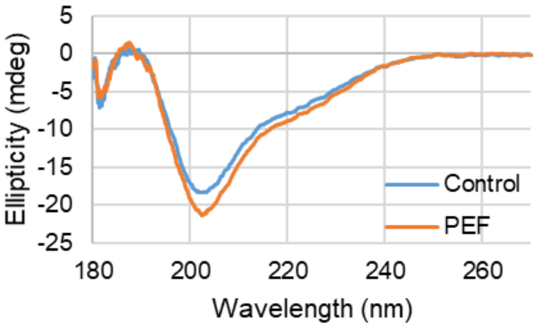

Figure S6. Circular dichroism spectra of non-PEF-treated (control) and PEF-treated NFDM. Measurements represent mean (*n*=3).

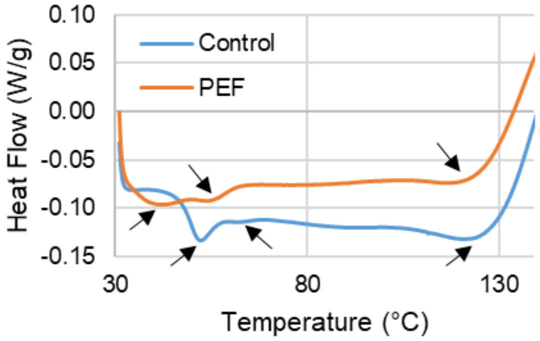

Figure S7. Differential scanning calorimetry thermograms of non-PEF-treated (control) and PEF-treated NFDM. Measurements represent mean (*n*=3).

Table S1. Mineral content (mg/mL) of the pellet (casein micelle) and supernatant (surrounding aqueous solution) of non-PEF-treated (control) and PEF-treated evaporated skim milk. Measurements represent mean ± standard deviation (*n*=3). ** signifies p < 0.01 and *** signifies p < 0.001 when comparing the PEF-treated measurement to the control.

**Treatment**	**Casein Micelle**			**Surrounding Aqueous Solution**		
	**Ca**	**P**	**Mg**	**Ca**	**P**	**Mg**
Control	6.59 ± 0.46	5.04 ± 0.02	0.52 ± 0.03	4.69 ± 0.38	5.11 ± 0.13	0.54 ± 0.01
PEF	3.55 ± 0.52**	3.93 ± 0.06***	0.35 ± 0.03**	6.63 ± 0.27**	6.09 ± 0.12**	0.62 ± 0.02**

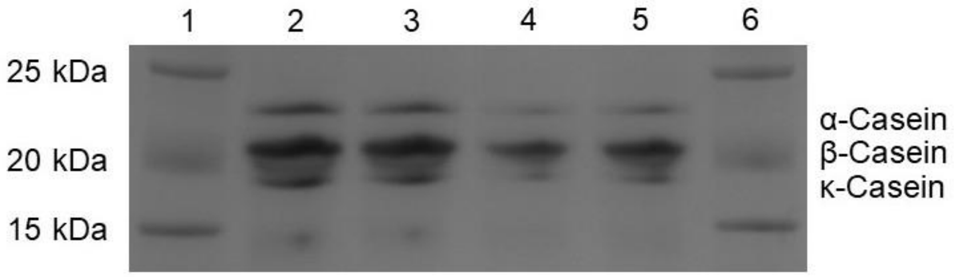

Figure S8. Molecular weight profile of the pellet (casein micelle) and supernatant (surrounding water) of non-PEF-treated (control) and PEF-treated evaporated skim milk. Lanes: 1 – protein standard ladder; 2 – control pellet; 3 – PEF pellet; 4 – control supernatant; 5 – PEF supernatant; 6 – protein standard ladder.

Table S2. PEF parameter combinations on 10% skim milk powder (SMP) used to determine the parameters used in this study.

**Blocks**	**Colinear Treatment Chamber**			**Parallel Treatment Chamber**		
	**Treat-ment**	**Field Strength (kV/cm)**	**Specific Energy (kJ/L)**	**Treat-ment**	**Field Strength (kV/cm)**	**Specific Energy (kJ/L)**
Block 1	Control	0	0	Control	0	0
	PEF	10	8, 15, 30	PEF	10	8, 15, 30
Block 2	Control	0	0	Control	0	0
	PEF	17	8, 15, 30	PEF	17	8, 15, 30
Block 3	Control	0	0	Control	0	0
	PEF	21	8, 15, 30	PEF	21	8, 15, 30
